# Epigenetic regulation of human FOXP3+ Tregs: from homeostasis maintenance to pathogen defense

**DOI:** 10.3389/fimmu.2024.1444533

**Published:** 2024-07-31

**Authors:** Yi Yue, Yuqing Ren, Chunya Lu, Ping Li, Guojun Zhang

**Affiliations:** Department of Respiratory and Critical Care Medicine, The First Affiliated Hospital of Zhengzhou University, Zhengzhou, Henan, China

**Keywords:** Tregs, epigenetics, FOXP3, immune homeostasis, tissue repair, metabolic regulation, hematopoiesis

## Abstract

Regulatory T cells (Tregs), characterized by the expression of Forkhead Box P3 (FOXP3), constitute a distinct subset of T cells crucial for immune regulation. Tregs can exert direct and indirect control over immune homeostasis by releasing inhibitory factors or differentiating into Th-like Treg (Th-Treg), thereby actively contributing to the prevention and treatment of autoimmune diseases. The epigenetic regulation of *FOXP3*, encompassing DNA methylation, histone modifications, and post-translational modifications, governs the development and optimal suppressive function of Tregs. In addition, Tregs can also possess the ability to maintain homeostasis in diverse microenvironments through non-suppressive mechanisms. In this review, we primarily focus on elucidating the epigenetic regulation of Tregs as well as their multifaceted roles within diverse physiological contexts while looking forward to potential strategies involving augmentation or suppression of Tregs activity for disease management, particularly in light of the ongoing global COVID-19 pandemic.

## 1 Introduction

The concept of Tregs originated in 1969 and 1970 when Nishizuka and Gershon discovered a subpopulation of thymic T cells capable of inhibiting the activity of other immune cells (1). The later discovery revealed that Tregs are a specific subset of CD4+ T cells, characterized by the presence of FOXP3, interleukin-2 receptor alpha (IL-2Rα), cytotoxic T lymphocyte antigen 4 (CTLA4), and other associated molecules. FOXP3, a member of the Forkhead transcription factor family, is among the key transcription factors governing Tregs development and function. IL-2R and CTLA4 can contribute to the suppressive functions of Tregs, partly through the induction of FOXP3 expression. Specifically, the combination of IL-2 with IL-2R activates the transcription factor STAT5, which in turn induces FOXP3 expression (2). On the other hand, CTLA4 modulates the interaction between CD28 and B7-1/B7 -2 on target cells’ surface to promote FOXP3 expression instead of inhibiting it (3). However, there is limited understanding regarding the synergy between CTLA4 and FOXP3, making it a potential direction for future research on Tregs. Therefore, the expression of FOXP3 plays an indispensable role in Tregs lineage commitment and maintenance, encompassing diverse functions such as proliferation, differentiation, survival, and apoptosis (4, 5). Under various physiological conditions, these cells play a crucial role in maintaining immune homeostasis, promoting tissue repair, regulating hematopoiesis, and ensuring metabolic balance (6, 7). Mutations in the *FOXP3* gene cause severe autoimmune diseases, tumor invasion, metastasis, and other catastrophic events such as infection-induced inflammatory storms (8–10).

The investigation of FOXP3+Tregs, particularly in terms of epigenetics, has become increasingly prominent in recent years (11). Our work elucidates the latest progress in the function and phenotype of Tregs in both healthy and pathological conditions (7, 12–15). The epigenetic alterations in Tregs, such as demethylation, histone modification and post-translational modification of *FOXP3*, confer optimal identity expression and inhibitory functionality upon these regulatory cells. Manipulation of *FOXP3* through epigenetic mechanisms to modulate the functionality of FOXP3+Tregs presents a promising avenue for the treatment of diseases. Treg-up strategies, such as targeting DNA methyltransferases (DNMTs), enhancing the function of ten-eleven translocation (TET) enzymes, and applying histone deacetylases inhibitors (HDACi), aim to increase the number or enhance the suppressive function of Tregs for treating autoimmune diseases, inflammatory diseases, transplant organ rejection, immune-related adverse reactions and overreaction to harmless antigens. In contrast, Treg-down approaches aim to reduce Tregs or their suppressive capacity to elicit antitumor immune responses and enhance pathogen defense (6). Furthermore, our work discusses the potential applications of FOXP3+Tregs in immune maintenance and pathogen defense during the global COVID-19 pandemic (16–18).

## 2 FOXP3+Treg regulates immune homeostasis through interactions with Th cells

**Figure 1:**
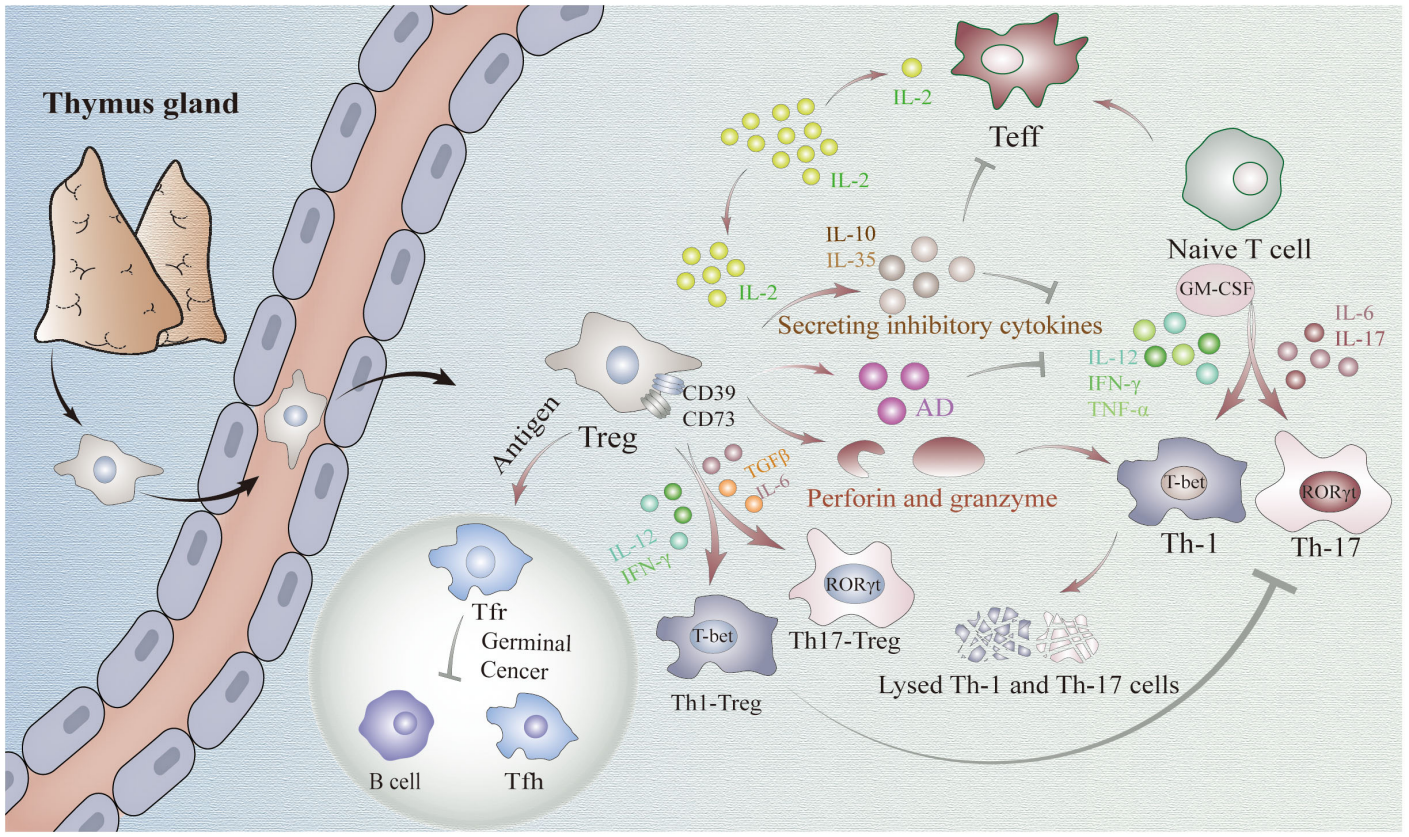
Mechanisms underlying the regulatory role of FOXP3+Tregs in immune homeostasis. Treg cells suppress the function of CD4+ effector cells through the release of inhibitory factors, such as IL-10 and IL-35. Additionally, they hinder Tconv activation by competitively consuming IL-2. Moreover, Tregs exhibit an inhibitory role in Th1, Th17, and other cell types by differentiating into Th-like Tregs or producing granzyme and perforin. Tconv cells: T conventional cells.

In order to counteract the invasion of exogenous pathogens, human have developed a robust and precise adaptive immune system centered around CD4+T helper cells, which play a pivotal role in defending against infections (19). Naive T cells can differentiate into Th1, Th2, Th17, Treg, and other cell subsets such as the follicle helper T (Tfh) and regulatory T follicular (Tfr) cells. Th1 and Th17 cells represent the principal effector CD4+T cell subsets in cellular immunity, responsible for mediating type 1 and type 3 immune responses, respectively (20–22). The former orchestrates host defense against intracellular pathogens such as bacteria and viruses, while the latter efficiently eliminates extracellular bacteria and fungi by recruiting neutrophils in a sustained manner. While, the germinal center(GC) of secondary lymphoid structures harbors Tfh cells as the principal effector subpopulation of CD4+T cells in humoral immunity. They are directly and indirectly influenced by Tregs, contributing to a sophisticated and intricate immune regulatory network within the body (**Figure 1**), as delineated below.

### 2.1 Treg inhibits Th1/17 by direct interactions

The function of Th1 cells relies on T-bet expression to produce IFN-γ and eliminate intracellular pathogens, thereby activating inflammatory responses (23). The concept of Th17 cells was first proposed in 2005 their unique capacity for secretion of the proinflammatory cytokine IL-17 at elevated levels (24). The co-presence of cytokines transforming growth factor beta (TGF-β) and IL-6 activates STAT3 and induces the transcription factor RORγt, which differentiates naive T cells into Th17 (25). However, in the presence of TCR(T-Cell Receptor), exposure to TGF-β alone induces upregulation of the transcription factor FOXP3 in naive T cells, thereby promoting their differentiation into Tregs (26). Although the specific mechanism remains elusive, we have discovered a partial correlation between the epigenetic inheritance of *FOXP3* below, which will be expounded upon later.

FOXP3+Tregs can inhibit the function of Th cells through various direct mechanisms. Firstly, Tregs can block the function of CD4+T effector cells by releasing a variety of inhibitory cytokines such as IL-10 and IL-35 (27). These cytokines reduce IL-12, IFN-γ, and TNF-α, thereby inhibiting Th1 cell differentiation (28, 29). Similarly, these cytokines also inhibit the differentiation of Th17 cells by suppressing IL-6 and IL-17 (29). Additionally, IL-10 reduces GM-CSF expression to impede the differentiation of both Th1 and Th17 cells, sand this effect can be potentiated by IL-35 (27). Secondly, Tregs are capable of rapidly sequestering IL-2 produced by T conventional cells (Tconv cells) during the early stages of immune response and prevent its further activation through competitive consumption (30). Thirdly, Tregs eliminate Th1 and Th17 cells through a killing mechanism mediated by granzyme and perforin (31). Finally, Tregs express extracellular enzymes CD39 and CD73 to promote adenosine production which inhibits cytokine production required for CD4+ effector T cell function while playing an anti-proliferative role (32). Despite thorough investigations into the existing mechanisms, the heterogeneity of Tregs remains poorly understood, leaving the possibility of undiscovered mechanisms yet to be explored in future research.

### 2.2 Tregs precisely regulates immune homeostasis by differentiating into Th-Tregs

Th1/17-like Tregs represent the predominant differentiated subset of Tregs during cellular immune responses. Exposure to inflammatory cytokines such as IL-12 or IFN-γ can drive the differentiation of human and mouse Tregs into Th1-like Tregs (33). Stimulation of human and mouse Tregs with TGFβ and IL-6 leads to upregulation of Th17 transcription factor, ultimately resulting in the generation of Th17-like Tregs (34). Under appropriate intensity of inflammatory stimulation, these Tregs can exhibit Th-like lineages with specific inhibitory function (35). Moreover, the upregulation of Th cell markers such as CXCR3 or CCR6 on their cells membranes enables more precise migration to the inflammatory tissue (36, 37). Consequently, they demonstrate a more selective inhibition of Th1/17 and CD8+T cell activation, thereby exerting a superior inhibitory role in Th1/17-related pathogenic immune response. This phenomenon has been validated in diabetes mellitus and crescent body nephropathy as well as other studies (12, 13).

In addition to cellular immunity, humoral immunity is a crucial component of the body’s immune homeostasis. B cells are the main cellular components mediating humoral immunity. Within the GC reaction, Tfh cells play a pivotal role in facilitating B cells production of high-affinity antibodies against antigens (38). A distinct subset of differentiated FOXP3+Tregs, known as Tfr cells, share surface markers with both Tfh and Tregs, thus representing a unique population situated between Tfh and Treg subsets referred to as Tfh-like Tregs (39, 40). It has been observed that exposure to antigens can induce the differentiation of Tregs into Tfh cells by up-regulating CXCR5 expression, enabling their migration towards GC where they ultimately acquire a follicular phenotype and assume the identity of Tfr cells (41). In addition to secreting granzyme and extracellular enzymes CD39 and CD73 (42), which can be also secreted by conventional Tregs, Tfr cells also possess other distinctive mechanisms of action for inhibiting Tfh and B cells: 1) inhibition of IL-1, CD80, and CD86 required for Tfh development and activation (43, 44); 2) production of neuritin and TNF-β to prevent abnormal accumulation of autoreactive B cells and Tfh cells in GC (45, 46); 3) down-regulation of IL-21, IL-4, IFN-γ, IL-10, and TNF-α expression in Tfh cells (39); 4) alteration of their own shape to impede contact between Tfh cells and B cells (47). Notably, a recent study discovered that Tfr also can promote the activation of B cells in GC possibly by suppressing the loss of B cells in GC through inhibition of aberrant cytotoxic Tfh cells with high expression levels of granzyme B and Eomes proteins (48).

Notably, it has been observed that the suppressive function of Th-Tregs is related to the intensity of inflammatory stimulation (33, 49, 50). Compared to pathogen-associated strong stimulation, Tregs exhibit stronger suppressive capabilities under weaker stimulation, such as self-antigens. This phenomenon is even more pronounced in Tfr cells due to their lower expression of CD25 (IL-2R) and the weaker competitive binding to IL-2 (51). Consequently, under IL-2 stimulation, Tfh cells activity is inhibited prior to Tfr cell activity, allowing better suppression of Tfh cell-mediated immune responses (51). However, under high concentrations of IL-2, Blimp-1 expression in Tregs is promoted, while Bcl-6, essential for normal Tfr cell development, is inhibited (51). This inhibition reduces the differentiation of Tregs into Tfr cells, facilitating the effective clearance of pathogens by effector B cells. Importantly, this process is reversible. when IL-2 levels decrease, Bcl-6 expression is upregulated, promoting the redifferentiation of Tregs into Tfr cells. This mechanism effectively prevents the accumulation of autoreactive B cell clones, indicating that Th-Treg differentiation can more precisely establish immune homeostasis (52).

Notably, the limitation of Tregs in regulating immune homeostasis should not be overlooked, as exceeding a certain threshold may result in immune dysregulation within the body. FOXP3 is likely to play a crucial role in Tregs differentiation towards Th phenotype (53). The potential mechanism lies in the fact that FOXP3 not only regulates the expression of IL-2-related STAT5 but also has been demonstrated to suppress the IL-6/STAT3 pathway mediated by RORγt *in vitro* studies (54, 55). Subsequently, it impedes the initiation of the Th17-Tregs differentiation program and restrains naive T cell differentiation into Th17 cells while promoting Tregs formation. And the expression of FOXP3 can be inhibited by inflammatory mediators in the local microenvironment (56). Considering its indispensable role in maintaining normal Tregs function and potential as a therapeutic target, we subsequently discuss the epigenetic regulation of *FOXP3*.

## 3 FOXP3+Treg maintains a variety of non-immune homeostasis

**Figure 2:**
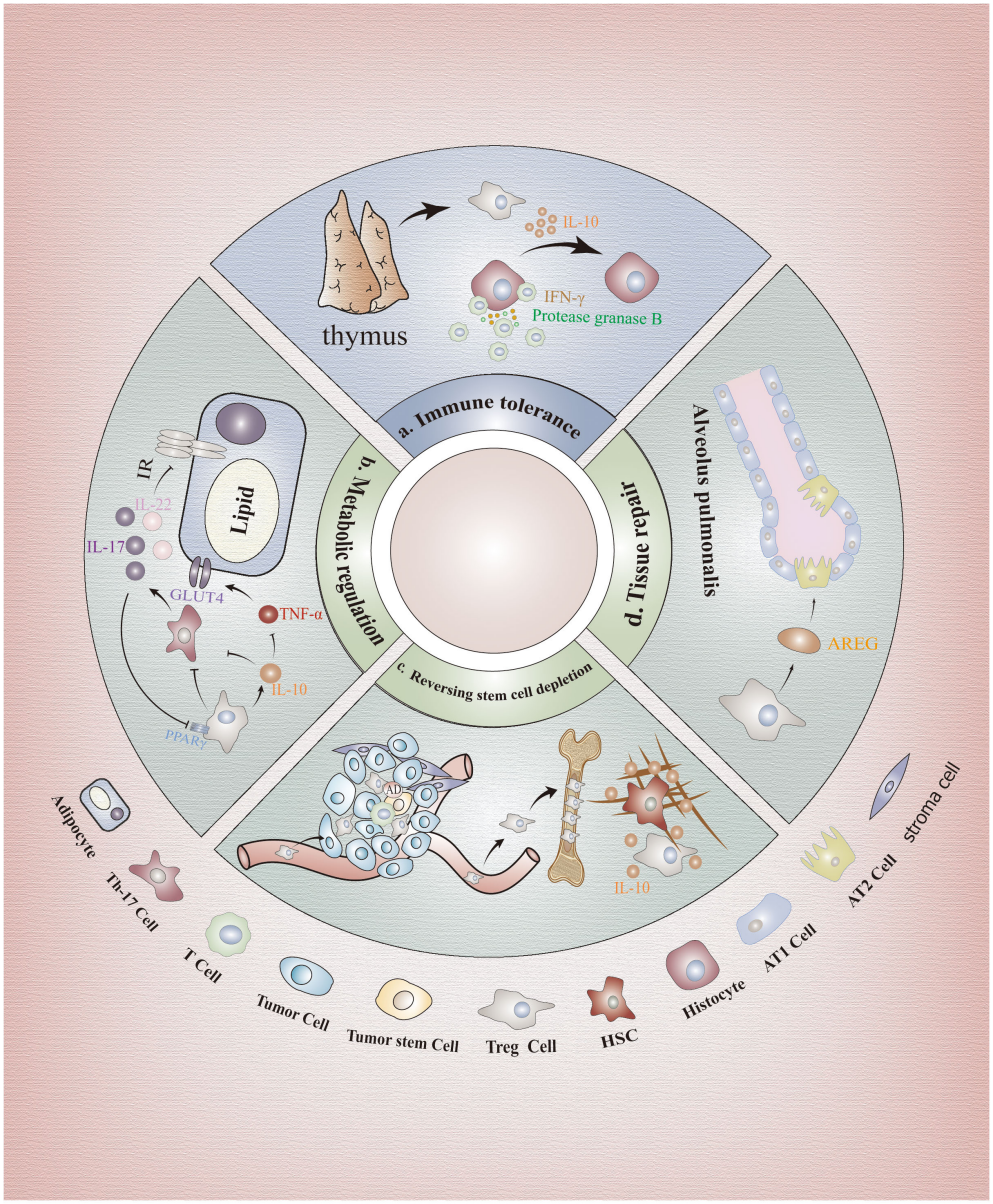
Immunological and non-immunological effects of FOXP3+Tregs. **(A)** Immunological effects: FOXP3+Tregs can protect tissue cells from Teff cells attack. **(B)** Regulation of metabolism: Tregs can suppress the expression of glucose transporter 4 through secretion of inhibitory factors, and impeding Th17 cells to alleviate the signaling pathway inhibition, enhancing adipocyte insulin sensitivity. **(C)** Maintenance of stem cells: IL-10 secreted by Tregs can preserve the self-renewal capacity of stem cell populations and prevent their depletion through regulation of stromal components. Furthermore, surface molecules CD39 and CD73 on Tregs also facilitate adenosine production, thereby subsequently safeguarding SCs against oxidative stress. **(D)** Tissue repair: AREG is a key factor in the process of tissue repair. Tregs in the lung promote the regeneration of type II alveolar epithelial cells by producing AREG. In addition, AREG is also found in muscles, neurons, skin and other tissues. HSCs, Hematopoietic Stem Cells; BM-Tregs, Bone Marrow Tregs; GLUT4, Glucose Transporter 4 Expression; AREG, Amphiregulin; ATII, Type II Alveolar Epithelium Cell.

In addition to the maintenance of immune homeostasis in the host microenvironment (**Figure 2A**), Tregs also play an important role in non-immune homeostasis such as stem cell maintenance, metabolism and tissue repair. Although nonimmune homeostasis does not encompass the recognition and response to bacterial, viral, or other pathogenic microbial invasions, it plays an equally pivotal role in upholding overall health and facilitating proper bodily functioning.

### 3.1 FOXP3+Treg prevents stem cells depletion

The specificity of Tregs was observed to be enriched in specific environments, such as the hematopoietic microenvironment in healthy individuals and the TME in cancer patients, making it one of the most representative features (57, 58). This phenomenon may be attributed to the interaction between high levels of CXCR4 expression on Tregs and elevated CXCL-12 expression on stromal cells within both the hematopoietic and tumor microenvironment, facilitating directed migration of Tregs towards these specialized niches (58–63). Consequently, these long-distance Tregs subsequently generate both analogous and distinct subsets in response to diverse microenvironments (57, 64, 65).

As previously mentioned, Tregs exert a potent immunosuppressive role. However, depletion of Tregs in the hematopoietic microenvironment did not result in increased proliferation of other T cells or production of proinflammatory cytokines, suggesting that immunosuppression may not be the primary function attributed to bone marrow Tregs (66). On one hand, IL-10 secreted by Tregs within the hematopoietic microenvironment prevents hematopoietic SCs exhaustion by modulating matrix composition while maintaining their self-renewal capacity (58, 66) (**Figure 2C**). On the other hand, CD39 and CD73 molecules expressed on Tregs in the hematopoietic microenvironment also facilitate adenosine production, thereby subsequently safeguarding hematopoietic SCs against oxidative stress stimulation (61). Notably, the aforementioned processes were also observed within the TME, indicating that Tregs exert non-immune functions in addition to their immune-related roles in TME. Additionally, recent *in vitro* and *in vivo* studies have revealed that the death of Tregs within the TME results in a substantial release of ATP, which is subsequently converted into adenosine by CD39 and CD73 to exert immunosuppressive effects surpassing those of live Tregs (32). This phenomenon not only hampers the clearance of tumor cells but also facilitates immune evasion by tumor cells. Due to the differential expression of CXCLs in stromal cells across various microenvironments, such as high expression of CXCL-12 in hematopoietic microenvironment and elevated levels of CCL17/22, CCL5, CCL1, and CCL28 in TME stromal cells, targeting the binding between different ligands and CXCR could serve as a promising therapeutic strategy (67–70). In addition, we can utilize the epigenetic regulation of Tregs, particularly the manipulation of methylation, histone modifications and post-translational modification of *FOXP3*, to abrogate its suppressive function while preserving its survival capacity for achieving anti-tumor efficacy. However, it is imperative to exercise caution and further research is warranted.

### 3.2 FOXP3+ Tregs increase insulin sensitivity and delay lipolysis

In recent years, accumulating evidence has demonstrated a correlation between glucose and lipid metabolic disorders and dysregulation of Tregs function (71, 72). Studies have shown that Th17 cells can impair insulin sensitivity and exacerbate insulin resistance by blocking the insulin receptor signaling pathway through enhanced secretion of IL-17 and IL-22, leading to metabolic dysfunction (73, 74). While Tregs can inhibit this process through various mechanisms closely related to the expression of FOXP3. Firstly, FOXP3 serves as the key transcription factor of Tregs, facilitating their ATP production and energy generation through the induction of oxidative phosphorylation (OXPHOS) and fatty acid oxidation (FAO) (75). Moreover, FOXP3 suppresses the differentiation of Th17 cells by directly interacting with RORγt and inhibiting its DNA binding activity (76). Besides, FOXP3 collaborates with PPARγ to confer naive CD4+T cell transcriptional characteristics resembling visceral adipose tissue-derived Tregs (77). Furthermore, FOXP3-expressing Tregs maintain insulin sensitivity in visceral tissues and regulate systemic metabolism by secreting IL-10 (78, 79). On one hand, IL-10 inhibits TNF-α function, downregulates glucose transporter 4 expression (GLUT4), and enhances adipocyte insulin sensitivity (75, 80) (**Figure 2B**). On the other hand, IL-10 suppresses IL-6 production while promoting the differentiation of naive CD4+T cells into FOXP3+Tregs rather than Th17 cells (80). Moreover, Tregs were found to secrete TGF-β, leading to a reduction in body weight and an improvement in insulin resistance, as demonstrated through experimentation on obese mice (81).

IL-17 reduces adipogenesis by downregulating the expression of specific proadipogenic transcription factors such as PPARγ (82). Cyclooxygenase-2 (COX-2) regulates Tregs proliferation in adipose tissue through negative feedback and can enhance the insulin-sensitizing effect of Tregs (83). Blocking COX-2 expression reduced the inhibitory effect of IL-17A on adipogenic differentiation (84). The perplexing finding is that a recent study indicates, contrary to previous results, that IL-10 derived from Tregs may drive insulin resistance in obese individuals by inhibiting the energy expenditure and thermogenesis of adipocytes (85). Depletion of the anti-inflammatory cytokine IL-10 enhances insulin sensitivity, mitigates diet-induced obesity, and induces Browning of white adipose tissue (86). This phenomenon may be attributed to the inhibitory effect of IL-10 on T cell differentiation into Th17 cells, thereby attenuating the down-regulation of PPARγ by IL-17 and ultimately leading to adipose tissue accumulation. However, the functionality of Tregs is more intricate than currently comprehended and is influenced by other cytokines within the local microenvironment. Future metabolic studies should delve into *Foxp3* epigenetics and interplay between multiple cytokines to substantiate this pathophysiological model.

### 3.3 FOXP3+ regulatory T cells participate in tissue repair

The involvement of Tregs in tissue repair has also been observed. It has been discovered that Tregs mainly contribute to tissue repair through the secretion of various growth factors, with amphiregulin (AREG) being identified as the pivotal factor in this process (72, 87). AREG has been extensively investigated in diverse tissues including muscle, neurons, skin, and lung. In skeletal muscle, Treg-derived AREG enhances blood flow restoration following hindlimb ischemia in mice by reducing apoptosis and accelerating histiocyte repair (88). Tregs also promote cardiomyocyte proliferation via paracrine signaling involving AREG (89). In UVB-irradiated skin, FOXP3+Tregs produce AREG to facilitate epidermal keratinocyte growth and maintain skin homeostasis (90). Recent studies have demonstrated that lung-resident Tregs produce the epidermal growth factor receptor ligand AREG, which promotes epithelial barrier regeneration and facilitates type II alveolar epithelium (ATII) cell regeneration for preserving lung function after respiratory viral infection while ensuring adequate blood oxygenation post-influenza virus infection (91) (**Figure 2D**). Furthermore, researchers have discovered that IL-33 activates downstream *Foxp3* via ST2 receptors to increase the abundance of Tregs in ischemic brain regions; subsequently leading to the production of AREG by these cells which then activates Epidermal Growth Factor Receptor (EGFR) located on neurons for enhanced recovery of brain cells (92).

Tregs have also been discovered to stimulate tissue regeneration through organ-specific recombinant regenerative factors, such as neurotropin-3 (NTF3), independent of IL-10, thereby highlighting a distinct role of Tregs beyond their conventional immunosuppressive function. Importantly, the induction of regenerative factors requires Foxp3, as Tregs lacking Foxp3 in damaged organs fail to express these factors (93). Additionally, FOXP3+Tregs have been identified to express keratin growth factors in the lung for maintaining tissue repair homeostasis (94).

## 4 The destiny of Tregs is mainly written by the epigenetic regulation of *Foxp3*

Despite the established significance of FOXP3 as a pivotal transcription factor enabling Tregs to exert their suppressive function, there remains some heterogeneity among the functionality of FOXP3+Tregs (95). The understanding of this phenomenon is intricately linked to the epigenetic mechanisms governing Tregs (96). The methylation, histone and the post-translational modification of *Foxp3* are crucial epigenetic regulatory mechanisms in Tregs. In particular, the Treg-specific demethylation regions (TSDRs) within the *Foxp3* gene, also known as CNS2, along with the other three conserved non-coding sequences (CNS0, CNS1 and CNS3), exhibits a hypomethylation pattern in mature Tregs (97–99). Therefore, understanding the unique hypomethylation patterns and post-translational modifications in these regions is crucial for comprehending the functional properties and phenotypic characteristics of Tregs.

### 4.1 Methylation of *Foxp3* gene

**Figure 3:**
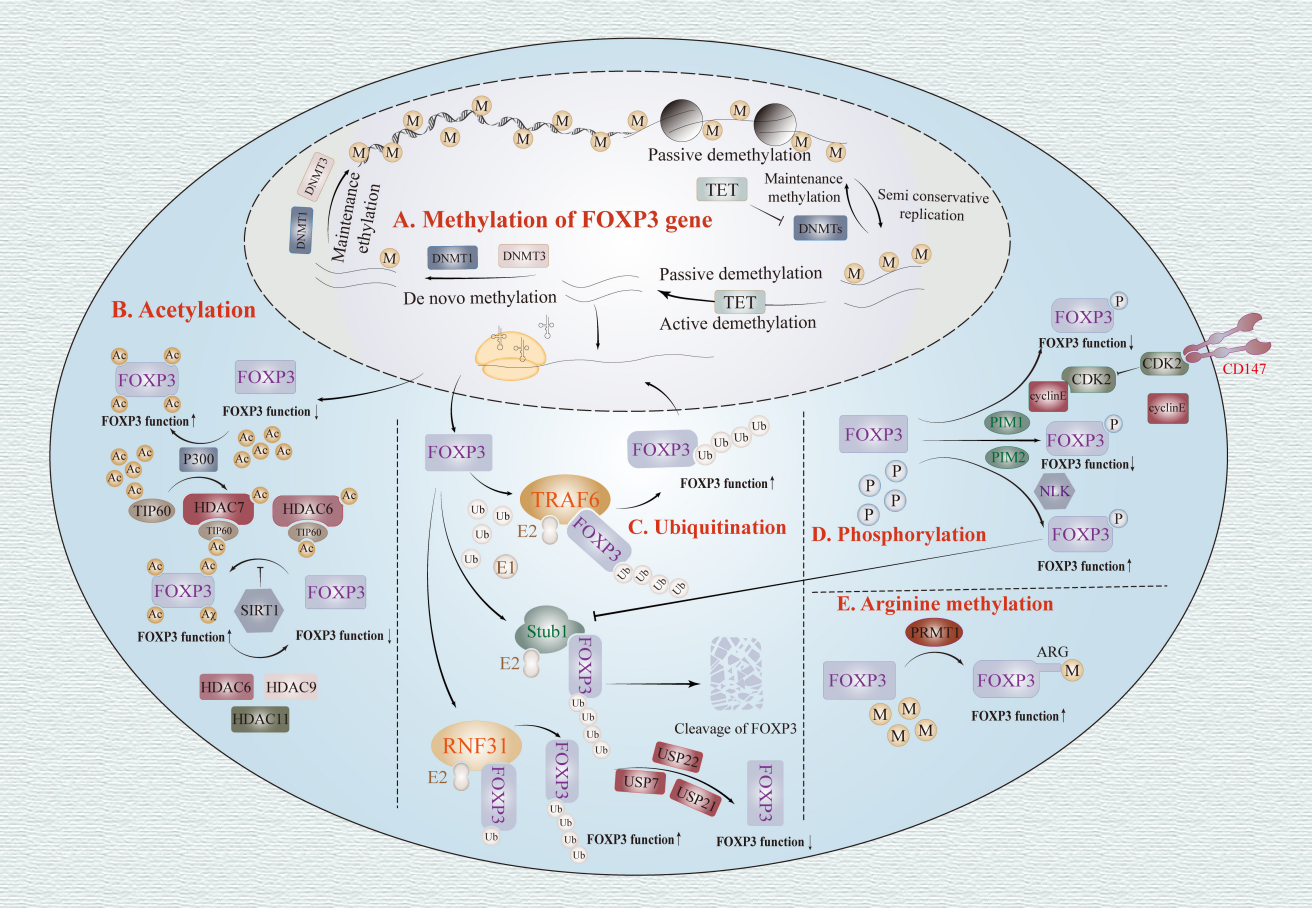
The epigenetic regulation of FOXP3. **(A)** Methylation: The methylation status of FOXP3 gene can modify the functionality of Treg cells. The methylated FOXP3 gene can modulate methylation levels through active demethylation facilitated by TET enzymes or partially retained replication during cell division, while also preserving methylation levels via *de novo* and maintenance methylation mediated by DNMTs. **(B)** Acetylation: TIP60 and P300 function as HATs, augmenting the activity of Tregs through facilitation of FOXP3 protein acetylation. Conversely, HDAC6/9/11 and SIRT1 exert negative regulation on FOXP3 acetylation, thereby inhibiting Tregs function. **(C)** Ubiquitination: The TRAF6 and RNF31 positively regulate the stability of FOXP3 protein and the function of Treg cells by catalyzing the ubiquitination of FOXP3 protein. STUB1 binds to HSP70 to mediate the down-regulation of FOXP3 and degrade FOXP3 protein through proteasomal degradation. Besides, the Enzymes, including USP7, USP21, and USP22, mediate FOXP3 deubiquitination, thereby stabilizing the function and plasticity of Foxp3+Treg. **(D)** Phosphorylation: The CDK2, PIM1, and PIM2 all impede the functionality of FOXP3+Treg by phosphorylating FOXP3. CD147, which is expressed on the surface of normal Treg cells, leads to the sequestration of CDK2, thereby inhibiting the degradation of FOXP3. NLK prevents STUB1-mediated ubiquitin-dependent protein degradation in Treg cells by phosphorylating FOXP3. **(E)** PRMT1 sustains the functionality of FOXP3+Treg cells by methylating arginine residues within the FOXP3 protein. Tregs, Regulatory T cells; FOXP3, Forkhead Box P3; TET, Ten-eleven Translocation; DNMTs, DNA Methyltransferases; HDACs, Histone Deacetylases; HATs, Histone Acetyltransferases; TIP60, Tat-interactive Protein 60; CoREST, RE1-Silencing Transcription Factor Corepressor; SIRT1, Sirtuin 1; RNF31, Ring Finger Protein 31; STUB1, Stip1 Homology and U-Box Containing Protein 1; TRAF6, TNF Receptor-Associated Factor 6; USP, Ubiquitin-Specific Protease; CDK2, Cyclin-Dependent Kinase 2; PP1, Protein Phosphatase 1; NLK, Nemo-Like Kinase; PRMTs, Protein arginine methyltransferases.

Methylation is a canonical regulatory pattern in epigenetics, predominantly occurring at DNA’s CpG sites (100). Alterations in its level can modulate the expression of genetic information by modifying the physical properties and spatial structure of DNA (101). Similarly, the methylation status of *Foxp3* gene influences genetic information expression in Tregs, which is dynamically maintained through DNA methyltransferase-mediated methylation coupled with active or passive demethylation induced by TET enzymes and cell mitosis (102) (**Figure 3A**).

#### 4.1.1 Restriction of FOXP3 expression by DNMT-dependent methylation

DNMTs, including DNMT1, DNMT3A, and DNMT3B, are the most extensively studied specialized enzymes involved in DNA methylation. Different types of DNMTs play distinct roles in maintaining *Foxp3* methylation (103).

DNMT1 is primarily responsible for maintaining the methylation of *Foxp3*, which requires the cofactor E3 ubiquitin ligase UHRF1(Ubiquitin-like with PHD and RING Finger domains 1) (104). Due to its high affinity for hemimethylated DNA and ability to recognize CpG sites of nascent strands characterized by short palindromic sequences, DNMT1 can precisely copy the methylation pattern on the former parental strand to the nascent strand, thereby inhibiting FOXP3 expression (105). Although complete knockdown of DNMT1 enhances apoptosis in Tregs, reduces the number and function of Tregs, and impairs the differentiation of Tregs precursor cells into FOXP3+Tregs, researchers have employed adeno-associated virus vectors carrying either DNMT1 or shDNMT1 under the control of the CD4 promoter (AAV-pCD4-shDNMT1 or AAV-pCD4-DNMT1) to achieve partial knockdown or overexpression of DNMT1, thereby modulating FOXP3 expression and manipulating Tregs function (106, 107). Hence, modulating the expression level of DNMT1 as a mean to regulate Tregs function may emerge as a promising strategy in future therapeutic interventions.

In addition, previous studies have demonstrated that Protein Phosphatase 6 (PP6) agonists and Signal Transducer and Activator of Transcription 6 (STAT6) inhibitors can augment the frequency of FOXP3 expression and enhance both the quantity and quality of Tregs (108, 109). The former mechanism disrupts methylation at *Foxp3* CpG sites by facilitating DNMT1 dephosphorylation (108, 109). Given that the latter is situated downstream in the JAK/STAT pathway, it is generally believed to possess superior targeting capabilities with fewer associated side effects (110). However, its impact on DNMT1 remains unclear and necessitates further investigation for potential therapeutic benefits in human diseases.

The semi-conserved duplication of genes facilitates the transmission of genetic information from parental cells to offspring cells. However, it has been proposed that higher levels of DNA methylation are not inherited through the semi-conservative replication machinery of mitosis, but rather established by *de novo* methylation catalyzed by DNMT3 (111). The DNMT3 family comprises mainly DNMT3A, DNMT3B, and DNMT3L, with the former two mediating *de novo* DNA methylation and the latter regulating their functions (112). Among them, DNMT3A plays a pivotal role in various adverse conditions characterized by high levels of inflammatory factors. It induces *de novo* methylation at the CpG site of *Foxp3* gene, leading to the loss of Tregs identity and differentiation into dysfunctional Tregs, thereby exacerbating immune dysfunction (113). Therefore, the differentiation of Tregs is likely to be predominantly mediated, at least in part, by DNMNT3A promoting *de novo* methylation that is dependent on *Foxp3*.

Since the catalysis by DNMT3A is crucial for the generation of novel methylation sites and increased levels of *Foxp3* methylation, in the absence of DNMT1, the passive demethylation process induced by cell mitosis will predominate (114). Additionally, while DNMT1 is capable of *de novo* methylation of *Foxp3*, its efficiency is lower compared to maintenance methylation (115). This highlights that efficient methylation of *Foxp3* requires the combined actions of both DNMT1 and DNMT3. Notably, similar to DNMT1, the activity of cofactor E3 ubiquitin ligase UHRF1 is also crucial for DNMT3 function. Targeted inhibition specifically against UHRF1 may provide novel therapeutic opportunities for autoimmune diseases without affecting Tregs survival.

#### 4.1.2 Demethylation of *Foxp3* by TET enzymes

The TET family of enzymes, including TET1, TET2, and TET3, catalyze the oxidation of Fe2+, Alpha-ketoglutaric acid (α-KG), glutaric acid or vitamin C to facilitate active DNA demethylation, converting 5-methylcytosine (5mC) into 5-hydroxymethylcytosine (5hmC) and other intermediates (116). CNS0-3, the *Foxp3*-specific super enhancer (SEs) in Tregs, are subject to regulation by TET enzymes, thereby playing a pivotal role in the transcriptional activation of the *Foxp3* gene (117, 118). Among them, CNS0 and CNS2 were the most important. The CNS0 initiates a transcriptional program specific to Tregs, which interacts with the TCR pathway to induce STAT5 activation upon IL-2 stimulation, thereby promoting TET3-mediated demethylation in this genomic region (2, 15). The CNS2, also known as TSDR, harbors a plethora of CpG islands characterized by well-established methylation patterns in mature Tregs, facilitating the stable expression of FOXP3 and thereby sustaining the distinctive features of Tregs (119). However, conditional deletion of TET2 and TET3 was observed to significantly restore DNA methylation on CNS2, implying that complete demethylation of CNS2 may rely on TET enzymes to counterbalance *de novo* DNA methylation catalyzed by DNMT3 (111, 120, 121). Collectively, it can be inferred that the expression of FOXP3 is partially attained through active demethylation of the *Foxp3* SEs facilitated by TET enzymes.

Moreover, due to its role in maintaining methylation, dysfunction of DNMT1 can lead to passive demethylation of the remaining copy of *Foxp3* on chromosomes, thereby potentially enhancing FOXP3 expression in Tregs through the function of DNMT1 (111). Deletion of DNMT1 induces apoptosis in FOXP3+Tregs, making the Developmental Pluripotency-Associated 3 (DPPA3) or Protein Arginine Methyltransferase 6 (PRMT6) protein capable of specifically targeting UHRFI a better alternative to direct knockdown of DNMT1 (122, 123). It is worth noting that the above process requires the involvement of TET enzymes (114). Additionally, under normal functioning conditions of DNMT1, TET oxidizes 5mC from *Foxp3* to 5-formylmethylcytosine (5fmC) and other oxidized-methylcytosines (oxi-mCs) that cannot be recognized by DNMT1 (124). This further facilitates the process of passive demethylation of *Foxp3*. Therefore, in order to enhance the Tregs state, it may be more advantageous to either counteract DNMT3-catalyzed DNA methylation *de novo* or induce DNMT1 function defect through the utilization of DPPA3 and PRMT6, while simultaneously augmenting the activity of TET enzymes.

*In vitro* studies have demonstrated the selective hydroxylation of specific sites for demethylation activation through genome editing techniques, such as fusion proteins of zinc-finger (ZF) or transcription activator-like proteins (TALEs) with TET family fusion proteins (125, 126). However, these approaches have been superseded by the clustered regularly interspaced short palindromic repeats-associated (CRISPR) system with deactivated Cas9 (dCas9), which offers enhanced potential for achieving complete demethylation. Recently, Aziz Taghbalout, Sumiyo Morita, and their colleagues have employed various CRISPR/dCas9 derivative technologies to precisely target TET1 to specific genomic loci, thereby implementing targeted activation sites for methylation (127, 128). This potential may empower individuals to overcome the adverse effects resulting from the non-specific demethylation of the entire genome caused by existing DNA methyltransferase inhibitors when addressing diseases characterized by specific epigenetic mutations, thereby potentially enhancing therapeutic efficacy in managing diseases with distinct epigenetic alterations. Despite the aforementioned advantages, complete control over CRISPR editing outcomes is still difficult to achieve (129). Moreover, most current gene editing procedures are conducted *in vitro*, posing challenges for efficient delivery of the gene editing system into an animal’s body and identification of smaller CRISPR editors (129). Nevertheless, its potential continues to positions it as a valuable contributor to advancements in healthcare, with the potentially offering new therapeutic approaches in the future.

### 4.2 Histone modification of *Foxp*3

**Figure 4:**
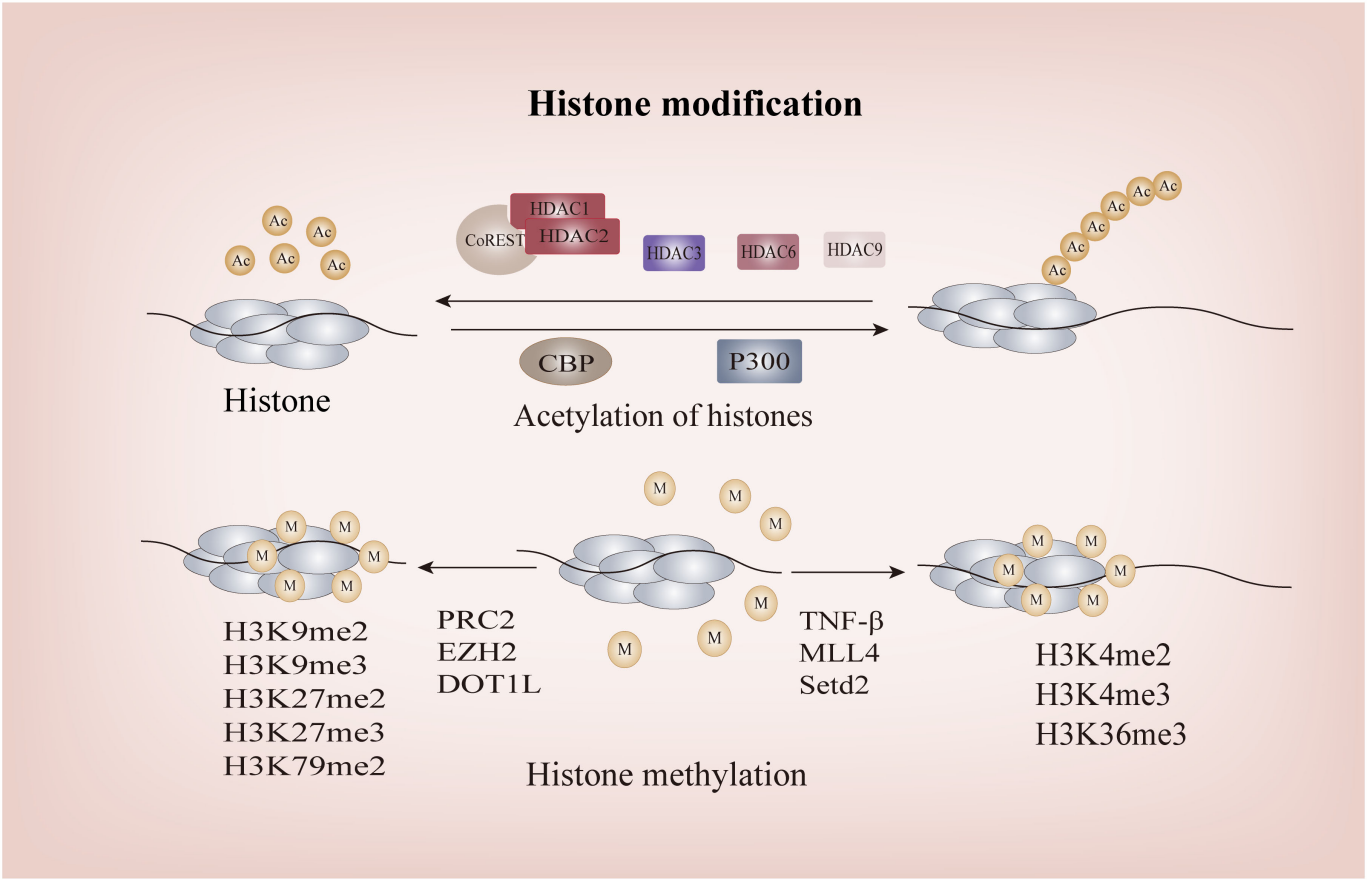
The Histone modification of *FOXP3*. P300 and CBP facilitate the acetylation of FOXP3, whereas the CoREST complex containing HDAC1 or HDAC2 impairs the functionality of FOXP3+Treg through deacetylation; Methylation of H3K4me2, H3K4me3, and H3K36me3 promotes FOXP3 transcription, while methylation of H3K9me2, H3K9me3, H3K79me2, H3K27me2, and H3K27me inhibits its transcription. HDACs, Histone Deacetylases; CoREST, RE1-Silencing Transcription Factor Corepressor; CBP, CREB-binding Protein.

After conducting an extensive investigation into gene methylation, it was determined that the expression of the *Foxp3* gene alone does not suffice to confer Tregs identity. The histone modification of the *Foxp3* locus also plays a crucial role in determining its fate (**Figure 4**). These modifications encompass acetylation, monomethylation, dimethylation, and trimethylation at various histone sites, thereby contributing to the intricate epigenetic mechanism.

Histone acetylation at the *Foxp3* loci initiates chromatin remodeling and transcription of FOXP3 by increasing their net negative charge, thereby disrupting their interaction with DNA and leading to the opening up of chromatin structure, ultimately enabling gene transcription and expression. As one of the classical Histone Acetyltransferases (HATs), *in vitro* studies have demonstrated that the interaction between CBP and the CNS2 region of *Foxp3* gene is a prerequisite for maintaining high levels of FOXP3 expression in Tregs under inflammatory conditions, thereby ensuring Tregs stability. This mechanism may involve CBP-mediated promotion of cyclic AMP response element binding protein/activating transcription factor (CREB/ATF), a transcriptional enhancer, to bind to the CNS2 region of *Foxp3*, consequently driving *Foxp3* transcription (130, 131). P300 exhibits a similar structure and biological function to CBP, both of which augment DNA binding capacity and regulate FOXP3+Tregs function. Simultaneous deletion of these enzymes results in the generation of a range of autoantibodies, underscoring their pivotal role as key regulators involved in *Foxp3* histone acetylation for preventing life-threatening autoimmune diseases (130). Additionally, histone acetylation of *Foxp3* can facilitate DNA demethylation in the CNS2 region of the *Foxp3* locus through TET involvement. This process eliminates the reliance of *Foxp3* transcription on histone tail acetylation at the promoter, thereby establishing a positive feedback loop to sustain *Foxp3* transcription (78).

The process of histone deacetylation is mediated by the HDAC family, comprising four distinct subfamilies: Class I (HDAC1, 2, and 3), IIA (HDAC6, 9), IIB (HDAC10), III (SIRT1), and IV (HDAC11). Notably, the research on subclasses IIB and IV primarily focuses on non-histone proteins and will be further elucidated in subsequent sections. RE1-Silencing Transcription Factor corepressor (CoREST) complexes, containing HDAC1 or HDAC2, impede the functionality of FOXP3+Tregs through deacetylation and augment the body’s anti-tumor capacity (132). Intriguingly, despite a significant increase in histone acetylation at the *Foxp3* promoter and CNS2 in HDAC3-/- mice as confirmed by Chromatin Immunoprecipitation assay, it resulted in detrimental autoimmune diseases (132). As this experiment does not exclude the impact of class IIA HDAC on FOXP3+Tregs and thus fails to fully elucidate the complex mechanism, targeting HDAC3 without compromising host Tregs function remains a challenge for researchers and clinicians. The development of potent and highly selective small-molecule inhibitors against class IIA HDACs may offer a potential solution. Furthermore, in the investigation of the tumor microenvironment(TME), it has been observed that Th17 cells possess the ability to recruit HDAC1, resulting in compaction of euchromatin within the proximal promoter region of CD73. This process effectively hinders its immunosuppressive properties. Conversely, Th17-Tregs derived from Tregs fail to recruit HDAC1, thereby enabling them to retain their immunosuppressive characteristics (133). This phenomenon may contribute significantly to tumor drug resistance Class IIa HDACs are exclusively expressed in lymphocytes. Through analysis of experimental databases, Keman Xu et al. discovered that HDAC6 can regulate the differentiation of Tregs into Th1-Tregs (134). This finding represents the first observation that histone deacetylation can modulate the plasticity of Tregs. However, it was observed that the number of FOXP3+Tregs in HDAC6-deficient mice remained unaltered and exhibited some retained suppressive functionality, thereby indicating that HDAC6 is not indispensable for the fate determination of Tregs, elucidating why complete knockout of HDAC6 does not lead to significant disease (135, 136). Similarly, HDAC9 can induce histone acetylation at the *Foxp3* loci, subsequently leading to reduced chromatin accessibility and consequent downregulation of *Foxp3* expression (137). SIRT1, the most representative and extensively studied enzyme among class III HDACs, has been demonstrated to play a crucial role in allograft renal transplantation mice. The study confirmed that pharmacological or genetic impairment of SIRT1 can prevent *Foxp3* histone acetylation, ultimately enhancing the inhibitory function of FOXP3+Tregs and improving the survival rate of mice undergoing allograft renal transplantation (138). In summary, HDACs primarily mediate acetylation modifications at the *Foxp3* loci, particularly in the promoter and TSDR/CNS2 regions, facilitating coordinated conversion of FOXP3 between chromosomes and chromatin to maintain genetic stability of FOXP3+Tregs.

Histone methylation can impact chromosome structure and protein binding affinity, thereby modulating gene transcription either positively or negatively (139). Notably, histone H3 is the most extensively methylated subunit, with lysine residues 4, 9, 27, 36, and 79 playing pivotal regulatory roles in gene expression (140). In contrast to histone acetylation, the specific functions of *Foxp3* histone tail are determined by the number and arrangement of methyl groups. H3K4me2, H3K4me3, and H3K36me3 normally facilitate *Foxp3* transcription, while H3K9me2, H3K9me3, H3K79me2, H3K27me2, and H3K27me3 inhibit its transcription (141–144). Stimulation of respiratory syncytial virus (RSV) leads to a significant increase in TNF levels in mice, resulting in the upregulation of H3K4 histone methyltransferase and subsequent methylation of *Foxp3* at three H3K4 sites within its promoter region, ultimately facilitating gene transcription (145). Similarly, Katarzyna Placek et al. demonstrated that the induction of FOXP3 expression necessitates methylation of H3K4, which is catalyzed by the MLL4 (Mixed Lineage Leukemia 4) protein (146). Furthermore, Setd2, a histone H3K36 methyltransferase, enhances the activity of *Foxp3* gene promoter and enhancer in Tregs while inhibiting their survival function (144). It is noteworthy that the sole upregulation of Nsd2, a histone H3K36 methyltransferase in Tregs, does not impact their differentiation and function, yet it plays a crucial role in promoting Tregs migration to the pregnant uterine decidua through enhancing CXCR4 expression. This novel mechanism deepens our understanding of methyltransferases (147). Initially, no compelling evidence of a significant association between the *Foxp3* promoter and H3K27me3 was observed (148). Subsequent investigations by Aaron Arvey and colleagues revealed that targeted enrichment of the *Foxp3* binding site in Tregs can effectively suppress H3K27me3, thereby enhancing Treg-mediated inhibition (149). The polycomb repressive complex (PRC), which catalyzes H3K27me3, can silence gene transcription, and homolog of zeste2 (EZH2), a histone methyltransferase of PRC2, is the key to histone H3 methylation at *Foxp3* promoter in Tregs (149). Although inhibiting EZH2 activity in Tregs can enhance the proinflammatory function of Tregs, alleviate the inhibition of Tregs on the recruitment and function of CD8+ and CD4+ effector T cells, and consequently reshape the TME, there is a potential risk of autoimmune diseases due to disruption in FOXP3-EZH2 interaction (150, 151). Therefore, while EZH2 holds promise as a future target, careful consideration must be given to avoiding potential adverse reactions. This remains one of the challenges faced by researchers in this field (151, 152).Additionally, recent studies have identified a potential mechanism involving the disruption of telomeric silencing 1-like (DOT1L) protein-mediated H3K79 methylation, leading to a reduction in FOXP3+Tregs (153). However, further investigation is required to fully elucidate this mechanism.

Lu et al. demonstrated *in vitro* and *in vivo* that all-trans retinoic acid (ATRA) enhances histone acetylation and hypomethylation in the *Foxp3* promoter region, thereby maintaining the expression of FOXP3 and the suppressive function of FOXP3+Tregs, while preventing their conversion into Th1 and Th17 cells (114). These findings underscore the pivotal role of histone modifications of *Foxp3* as determinants of Tregs fate, highlighting the need for future investigations on epigenetic mechanisms governing histone modifications in FOXP3+Tregs to gain deeper insights into Tregs lineage commitment and identify potential targets for novel therapeutic strategies.

### 4.3 Post-translational modification of FOXP3 protein

In addition to the post-translational modifications of histones in the *Foxp3* genome, various post-transcriptional modifications including acetylation, phosphorylation, ubiquitination, and methylation regulate the function, stability, and subcellular localization of FOXP3. These modifications significantly contribute to the regulation of FOXP3 expression and determine Tregs fate by influencing the differentiation, survival, function, activity, and stability.

#### 4.3.1 Acetylation

The acetylation of the FOXP3 protein hinders its K48 polyubiquitination and subsequent degradation by the proteasome (154). The post-transcriptional acetylation level of FOXP3 is mainly determined by HATs in conjunction with HDACs (**Figure 3B**). In Tregs, there are three HATs to accomplish acetylation of FOXP3 protein, while the deacetylation is mainly regulated by nine HDACs. TAT-interacting protein (TIP60) and p300, as the HATs, regulate the acetylation of FOXP3, with TIP60 being the primary and extensively investigated HAT in this particular context (155). TIP60 can form complexes with HDAC7 or HDAC9, thereby modulating the acetylated FOXP3 function and promoting transcriptional inhibition mediated by FOXP3 (155, 156). The study of rheumatoid arthritis(RA) revealed that diminished levels of TIP60 disrupt the acetylation process protecting the FOXP3 protein, leading to impaired functionality of FOXP3+Tregs and promoting Th17 differentiation (157). Furthermore, TIP60 can facilitate K327 acetylation through its interaction with P300, thereby promoting optimal FOXP3 post-translational acetylation activity and augmenting the functionality of Tregs (158). These findings enhance our comprehension of Tip60’s role in post-translational modification of the FOXP3 protein and present a novel target for future modulation of Treg-DOWN or UP.

HDACs can exert their enzymatic activity not only on histones, but also on non-histone proteins. The deacetylation of FOXP3 is mediated by a panel of HDAC isoforms including HDAC6, 9, 10, 11 and Sirt1 (137, 159–162). Although HDAC6 has been implicated in the regulation of Tregs plasticity and heterogeneity through histone deacetylation as mentioned above, recent studies have demonstrated its predominant localization in the cytoplasm and its deacetylase activity primarily targeting non-histone substrates such as FOXP3 (159). This is shared by the class IIB histone deacetylase HDAC10 and the distinct class IV histone deacetylase HDAC11, all three possess the ability to selectively deacetylate lysine residues on FOXP3 protein without compromising the integrity of healthy cells, thereby preserving the suppressive function of Tregs (159–161, 163–165). Currently, selective HDAC6 inhibitors, such as ACY241, are regarded as the most promising and appealing therapeutic approach (166, 167). Despite exhibiting no apparent toxic or side effects, unexpected complications such as thrombosis, thrombocytopenia, and neutropenia have been observed in empirical studies due to the limited selectivity of drugs (168). Therefore, it remains imperative to overcome off-target effects of drugs in order to further enhance drug targeting accuracy. Differing from traditional HDACs, SIRT1 exerts a negative regulatory effect on the acetylation of FOXP3 by inhibiting TIP60 self-acetylation. Despite the concurrent increase in FOXP3 protein acetylation, the absence of SIRT1 does not impact the functionality of effector T cells (162, 169, 170). This observation holds potential for providing novel insights to researchers grappling with the challenge of enhancing anti-tumor immune effects without exacerbating immunorelated adverse reactions.

In conclusion, despite the need for further investigations into the acetylation of FOXP3 protein, the epigenetic modulation of FOXP3 acetylation and deacetylation still holds significant potential for therapeutic interventions in immune-related disorders.

#### 4.3.2 Ubiquitination

Among more than 200 post-translational modifications, ubiquitination is one of the most prevalent and the most intensively studied regulatory modifications. Ubiquitin modifications can consist of monomers or polyubiquitin chains. This process is carried out by a combination of ubiquitin activating enzymes (E1), ubiquitin conjugating enzymes (E2), and ubiquitin ligases (E3). The ubiquitin-activating enzyme E1 is highly conserved and able to activate ubiquitination by expensing ATP. Ubiquitin-conjugating enzyme (E2), which connects E1 and E3, is also known as ubiquitin transporter. Ubiquitin ligase E3 determines the specific recognition of target proteins and is considered to be the most critical enzyme in the ubiquitination process (171). In Tregs, Ring Finger Protein 31 (RNF31), Stip1 Homology and U-Box Containing Protein 1 (STUB1), and TNF Receptor-Associated Factor 6 (TRAF6) are the E3 ligases that directly ubiquitinate FOXP3 (**Figure 3C**). The TRAF6 protein has been demonstrated to exert a stabilizing effect on IL-2 expression and an inhibitory effect on Th-17 differentiation, possibly due to TRAF6-mediated K63-linked ubiquitination that ensures appropriate localization of FOXP3 (172, 173). RNF31 can positively regulate the stability of FOXP3 and the function of Tregs by catalyizing the binding of atypical ubiquitin chain to FOXP3 protein. Knockdown of RNF31 in human Tregs reduced FOXP3 protein levels and increased interferon-γ levels, leading to Tregs differentiation towards a Th1 helper cell-like phenotype (174). Unlike the first two enzymes, STUB1 can bind to Heat Shock Protein 70 (HSP70) to mediate downregulation of FOXP3 and then degrade FOXP3 via the proteasome (175, 176).

Enzymes involved in deubiquitination, including Ubiquitin-Specific Protease 7/21/22 (USP7/21/22), have been shown to stabilize the function and plasticity of FOXP3+Tregs. USP7, which is observed to be decreased in non-obese diabetic mice, decreases FOXP3 polyubiquitination and increases FOXP3 expression, and this process is thought to be achieved by promoting the multimerization of TIP60 and FOXP3 (177–179). USP21 have the ability to prevent FOXP3 depletion at the protein level and restrict the generation of Th1-Tregs (180). In various cancer models in mice, USP22 specificity ablation has been demonstrated to impede tumor growth by reducing FOXP3 protein levels (181). Moreover, both the USPs can effectively modulate TGF-β and specific stressors, such as oxygen induction, to optimize their functionality (182). These findings provide support for the involvement of ubiquitination and degradation of FOXP3 protein in the regulation of suppressive function exhibited by Tregs, thereby suggesting that targeting the ubiquitination process of this protein holds promise for modulating immune homeostasis.

It is noteworthy that the interplay between TRAF6, USP7, and low levels of STUB1 has been implicated in enhancing the stabilization of TSDR demethylation for FOXP3 expression (183). The regulatory function of Tregs operates through a multifaceted network involving diverse modification processes in transcription and translation, which can easily be overlooked. Therefore, future research on FOXP3+Tregs epigenetics should place greater emphasis on exploring different modes of epigenetic crosstalk.

#### 4.3.3 Phosphorylation

Cyclin-Dependent Kinase 2 (CDK2), Protein kinase PIM1 and PIM2 all inhibit FOXP3+Tregs function by directly phosphorylating FOXP3(**Figure 3D**). However, they exert differential regulation on FOXP3 phosphorylation through distinct mechanisms. CDK2 potentially exerts a negative influence on the stability and activity of FOXP3+Tregs by phosphorylating Ser-19 and Thr-175 residues on the FOXP3 protein, with the specific mechanism likely involving phosphorylation-dependent ubiquitination (184). Additionally, during the investigation of CD147, a transmembrane glycoprotein expressed on the surface of normal Tregs, it was observed that the intracellular domain of CD147 can form an association with CDK2, resulting in sequestration of CDK2. Consequently, this interaction inhibits FOXP3 phosphorylation and prevents degradation of FOXP3 (185). The activation of CD147 is facilitated by the ubiquitously expressed membrane protein CD98, indicating that targeting the CD98-CD147-CDK2 interaction may hold therapeutic potential for immune-related disorders. Although direct evidence for the reduction of FOXP3 protein expression by protein kinase PIM1 was not found, it exhibited the ability to impair the chromatin binding activity of FOXP3 protein (186). Furthermore, increased expression of the proinflammatory cytokine IL-6 can promote PIM1 expression and ultimately lead to Tregs differentiating into effector T cells, raising the possibility that Pim1-mediated phosphorylation of FOXP3 protein is responsible for naive T cells being able to differentiate into Tregs when exposed to TGF-β alone, but not together with IL-6 (26, 186). In contrast to these enzymes, Nemo-Like Kinase (NLK) upholds Tregs function through phosphorylation of FOXP3. Specifically, NLK-mediated phosphorylation of FOXP3 stabilizes FOXP3 protein levels by inhibiting STUB1-mediated ubiquitin-dependent protein degradation in Tregs. The pivotal step for initiating this process involves the activation of TGF-β-activated kinase 1 (TAK1) (187). These observations highlight the intricate interplay between ubiquitination and phosphorylation, thereby warranting further investigation into the crosstalk among diverse epigenetic modalities in future studies.

#### 4.3.4 Arginine methylation

Arginine methylation of FOXP3 protein maintains Tregs fate through seemingly paradoxical effects (**Figure 3E**). Protein arginine methyltransferases (PRMTs) have emerged as promising therapeutic targets, with PRMT1 and PRMT5 being two essential enzymes for FOXP3 protein methylation. PRMT1 can methylate FOXP3’s arginine residues at positions 48 and 51, and inhibiting methylation specifically at these sites attenuates the suppressive function of FOXP3+Tregs, highlighting the importance of PRMT1 in maintaining their activity (188). Similarly, PRMT5 also plays a crucial role in Tregs suppression, as demonstrated by severe autoimmunity observed in PRMT5 conditional knockout mice (189). Furthermore, studies suggest that the complex formed by PRMT1 and RORγt promotes Tregs differentiation into Th17 cells in mice while PRMT5 enhances DNMT1’s binding to the *Foxp3* promoter limits Tregs differentiation (190, 191). Moreover, it is this seemingly paradoxical function that is critical for Tregs to maintain homeostatic balance.

## 5 Therapies based on epigenetic manipulation of FOXP3+ Tregs: autoimmune diseases and tumors

### 5.1 The Treg-up strategy protect against autoimmune diseases

Autoimmune diseases (AD) are significant contributors to the burden of chronic illnesses globally. These conditions, such as RA, systemic lupus erythematosus (SLE), inflammatory bowel disease (IBD), and autoimmune hepatitis, present substantial challenges for both patients and clinicians alike. Although not universally prevalent, the incidence of these diseases is on the rise. Despite exhibiting distinct clinical manifestations, they share a common pathogenesis characterized by aberrant humoral and cell-mediated immune mechanisms (192–195).

**Figure 5:**
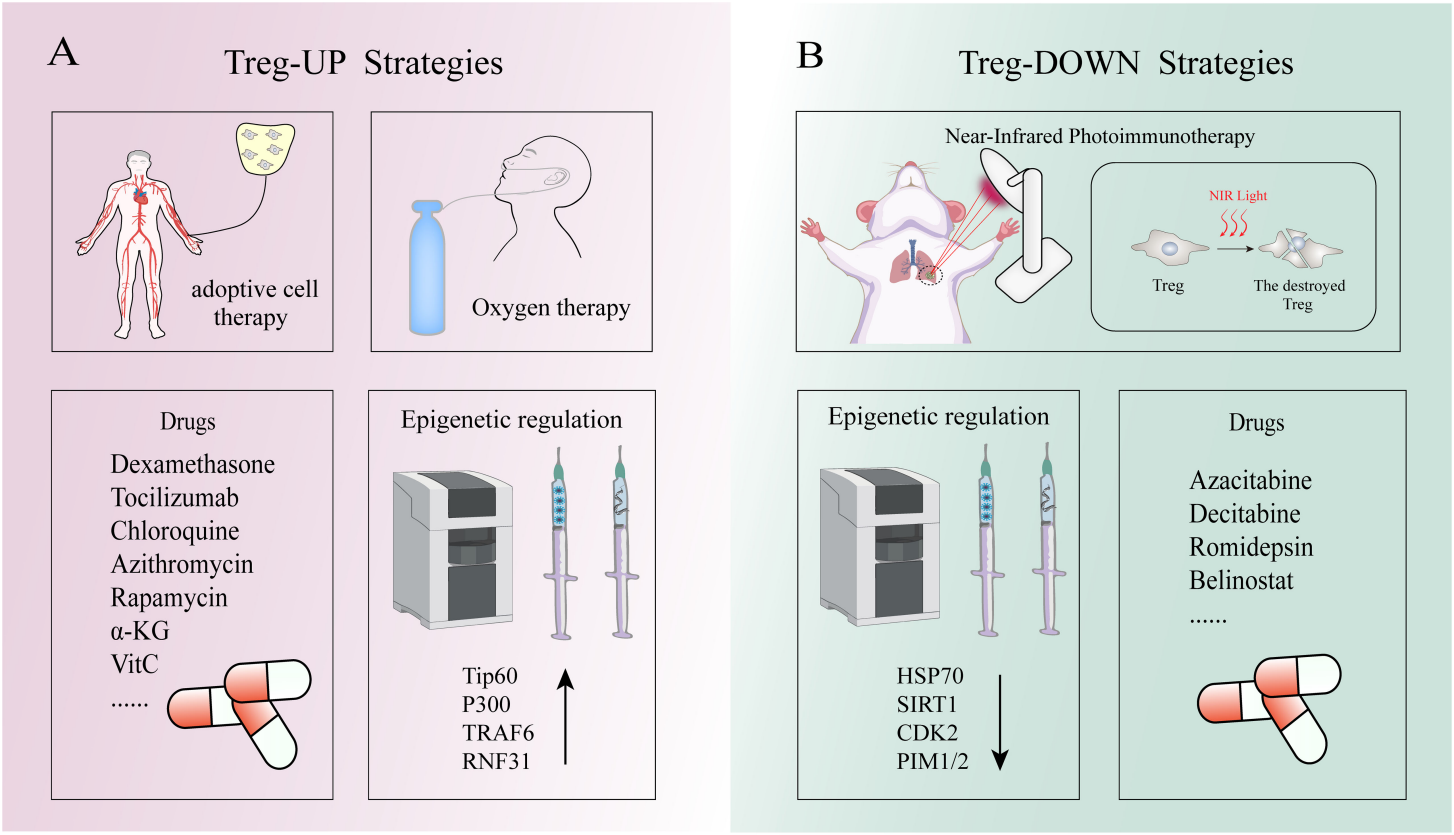
The Strategies of Treg-up and Treg-down. **(A)** Treg-up strategies: Oxygen therapy can prevent hypoxia inducing factor 1 alpha mediated Proteasome to the degradation of FOXP3, thus maintain Tregs inhibition function. Low-dose IL-2, as well as medications such as dexamethasone, tocilizumab, chloroquine, and azithromycin, can upregulate FOXP3 expression in Tregs. TIP60 and p300 can modulate the acetylation of FOXP3 to uphold the functionality of Treg cells. TRAF6-mediated K63-linked ubiquitination ensures the accurate subcellular localization of FOXP3. RNF31 positively regulates the stability of FOXP3 and enhances the functionality of regulatory T cells by catalytically binding the non-canonical ubiquitin chain to FOXP3 protein. **(B)** Treg-down strategies: Under near-infrared (NIR) light irradiation, the application of light-activated antibodies selectively enables depletion of Treg cells within tumor tissues. The interaction between STUB1 and HSP70 promotes the proteasomal degradation of FOXP3 protein. Additionally, the phosphorylation of FOXP3 by CDK2, PIM1 and PIM2 impedes the functionality of FOXP3+Tregs.

Treg-based therapies are potential in controlling or curing autoimmune diseases (**Table 1**). The most promising immunosuppressive strategy for autoimmune diseases is Treg-UP, which involves in adoptive cell therapy of Tregs, vivo expansion of natural Tregs and stabilization of their suppressive function (**Figure 5A**) (6). Given that adoptive therapy does not fall within the realm of epigenetics, it will not be expounded upon in this context. *In vivo* expansion of native Tregs is an effective strategy because these cells express high-affinity IL-2 receptors and are highly sensitive to changes in IL-2 levels (30). Using low doses of IL-2 can induce Tregs expansion *in vivo* while avoiding the inflammatory response caused by IL-2 signaling on NK cells or effector T cells (196, 197). By inhibiting the interaction between IL-2 and IL-2Rβ as well as IL-2Rγ on Tconv cells has also been shown to successfully expand the number of Tregs in patients with autoimmune diseases (198).

**Table 1:** List of the current research studies focusing on Tregs for autoimmune diseases.

Conditions	Interventions	Identifier	Status
Autoimmune Diseases	Treg cells	NCT02704338	PHASE1/2
Type1 Diabetes Mellitus	Liraglutide + UCB-Tregs	NCT03011021	PHASE1/2
Ulcerative Colitis	Treg cells	NCT04691232	PHASE1
Autoimmune Hepatitis	Low Salt Diet	NCT02050646	NOT APPLICABLE
Type1 Diabetes Mellitus	UCB-Tregs +Insulin	NCT02932826	PHASE1/2
Autoimmune Diseases	IL-2	NCT01988506	PHASE2
Rheumatoid arthritis	Tocilizumab + Adalimumab	NCT02963402	NOT APPLICABLE
Type 1 Diabetes Mellitus	Ex vivo Expanded Human Autologous Polyclonal Treg cells	NCT01210664	PHASE1
Type 1 Diabetes Mellitus	PolyTregs+IL-2	NCT02772679	PHASE1
Systemic lupus erythematosus	Ex vivo Expanded Human Autologous Polyclonal Treg cells	NCT02428309	PHASE1
Extramembranous Glomerulopathy	Blood sample	NCT05428605	NOT APPLICABLE
Multiple Sclerosis	Nanocurcumin	NCT03150966	PHASE2
Multiple Sclerosis	Hookworm larvae	NCT01470521	PHASE2
Rheumatoid Arthritis	SBT777101	NCT06201416	PHASE1
Systemic Lupus Erythematosus	Low-dose IL-2	NCT03312335	PHASE2
Type 1 Diabetes	Low-dose IL-2	NCT02411253	PHASE2
Neuromyelitis Optica	Peripheral blood	NCT05245344	PRECINICAL

UCB-Tregs, Umbilical Cord Blood Treg cells.

However, simply increasing the number of Tregs without considering their stability does not yield the desired results in treating autoimmune diseases. In recent years, stable Tregs function has been recognized as a crucial factor in such treatment. Prior to the development of epigenetic techniques, scientists induced stable FOXP3 expression in Tregs by inhibiting Protein Kinase B-mammalian Target of Rapamycin (AKT-mTOR) signaling pathway activation with rapamycin and blocking T cell Receptor-Cyclin-Dependent Kinase (TCR-CDK) pathway activation with CDK8/19 inhibitors (38, 199). As mentioned earlier, active or passive demethylation by TET enzymes maintains hypomethylation of *Foxp3*’s TSDR, which plays a critical role in suppressing Tregs function for optimal treatment of autoimmune diseases (200, 201). This effect is enhanced by α-KG and vitamin C or ascorbic acid. The targeted inhibition of UHRF1 by DPPA3 or PRMT6 proteins may mitigate the suppressive impact of DNMTs on FOXP3+Tregs, thereby offering promising prospects for the treatment of autoimmune diseases (122, 123). Additionally, post-transcriptional epigenetic modifications such as acetylation, phosphorylation, ubiquitination and methylation of FOXP3 can also regulate its expression and stabilize optimal suppressive function (8).

Although the emergence of epigenetics has brought hope for curing patients with autoimmune diseases, the non-specific manipulation of epigenetic effectors (such as non-selective HDAC inhibitors) has failed to yield the desired therapeutic outcomes. However, CRISPR-dCAS9 can be utilized as an ideal method to precisely target epigenetic effectors. Currently, various epigenetic effectors have been employed to design and synthesize sgRNA-dCas9 circuits, demonstrating favorable outcomes (8). Nevertheless, a comprehensive genome-wide map regarding off-target binding of sgRNA-dCas9 is lacking, along with knowledge about factors controlling off-target binding and understanding of the molecular/cellular environment conducive to off-target effects. Therefore, the off-target effect of sgRNA-dCas9 remains a major drawback in its application for basic and translational research in prokaryotic and eukaryotic systems. Fortunately, deep learning techniques have recently been applied and show promise in overcoming the challenge of sgRNA design (202).

### 5.2 The Treg-down strategy targeting FOXP3 presents a promising avenue for conquering malignancies

The therapeutic strategies targeting the TME have emerged as a promising approach for cancer treatment due to its dystrophic, lactate-rich hypoxic characteristics that significantly impact tumor growth, metastatic dissemination, and response to therapy (**Table 2**). The TME encompasses diverse cellular populations including cancer cells, endothelial cells responsible for vasculature formation, lymphocytes and macrophages involved in immune surveillance, and fibroblasts constituting the extracellular matrix (203). Among them, Tregs infiltration in the TME has been observed to be elevated across various human tumor types. In addition to their role in preventing SCs depletion as mentioned above, Tregs generally exhibit a lower rate of glucose consumption and demonstrate relative tolerance towards elevated lactate concentrations compared to effector T cells (204, 205). This unique metabolic adaptation enables them to sustain their functionality within a hypermetabolic environment while minimizing competition with effector T cells for maintaining immunosuppressive activity. Down-regulation of Tregs attenuated anti-tumor immune responses and inhibited tumor invasion. This phenomenon can be attributed to FOXP3-mediated metabolic reprogramming of Tregs, in which the AMP-activated protein kinase (AMPK) and mTOR signaling pathways are involved (206). Notably, it has been previously mentioned that Tregs death may facilitate immune evasion by cancer SCs. Therefore, the “Treg-down” strategy primarily focuses on epigenetic modulation of Tregs to attenuate the differentiation of naive T cells into Tregs within the TME, impeding peripheral migration of Tregs to the TME or suppressing their functional activity rather than disrupting their survival (**Figure 5B**). Furthermore, since most human solid cancers are immune cold tumors that do not respond well to immunotherapy alone, epigenetic drugs can induce ICD to convert cold tumors into hot ones thereby achieving better anti-tumor effects. Epigenetic tools, including DNMTs, HDACs and their inhibitors, have been utilized to manipulate Tregs and enhance the anti-tumor ability of host immune cells. DNMTi such as Azacitabine and Decitabine, and HDACi such as Romidepsin and Belinostat have shown promising results in the clinical treatment of human malignant tumors (207–210). However, despite some success in treating certain types of solid tumors including T-cell lymphomas, epithelioid sarcomas and refractory follicular lymphomas with epigenetic agents alone, they have not yet been sufficiently effective in advanced tumors to obtain FDA or other regulatory approval, which need further study. Additionally, there is a belief among certain individuals that the specificity of epigenetic regulators in conjunction with conventional immune therapies (such as cancer vaccines, ICIs, soluble tumor viruses, CAR-T cells and other novel immunostimulants) could potentially enhance the therapeutic efficacy through a rational combination strategy (211). However, many tumor-associated antigens are autologous or quasi-autologous, meaning that successful antitumor immunity through Tregs depletion often leads to autoimmunity (212). Therefore, the primary challenge in Treg-depleted tumor immunotherapy lies in precisely targeting tumor-infiltrating Tregs(TI-Tregs) within the TME, while preserving Tregs in normal tissues to uphold immune autotolerance and homeostasis. Previous studies have demonstrated that light-activated antibodies targeting Tregs can selectively deplete these cells within tumor tissues upon near-infrared (NIR) light exposure (213). Furthermore, researchers have discovered that the adaptive metabolism of tumor-infiltrating TI-Tregs in the TME could serve as a potential target for cancer therapy due to their ability to enhance ATP production under hypoxic conditions through FOXP3-induced OXPHOS (214). Therefore, employing epigenetic techniques to inhibit the adaptation of TI-Tregs to low glucose, high lactate, and high lipid environments may represent a more effective strategy for overcoming tumor immune evasion. In summary, scientists increasingly recognize the significance of employing a “Treg-down” approach in conquering cancer and emphasize the need for precise strategies targeting future TI-Tregs.

**Table 2:** List of the current research studies focusing on Tregs for tumor treatment.

Conditions	Interventions	Identifier	Status
Leukemia Hematologic Malignant Neoplasms	Infusion of Tregs High dose irradiation conditioning + Tregs	NCT02991898 NCT03977103	PHASE2 PHASE2
Myeloproliferative Neoplasms	Ipilimumab+ CD25hi Treg depleted DLI	NCT03912064	PHASE1
Hematologic Malignancies	CD25/Treg-depleted DLI	NCT00675831	PHASE1
Hematological Malignancies	Treg depleted DLI	NCT06180499	PHASE1/2
Hematological Malignancies	Treg depleted DLI	NCT03236129	PHASE3
Leukemia	UCB transplantation + Total body irradiation + Treg infusion + Anti-tumor drugs	NCT00602693	PHASE1
Stem Cell Transplant Complications	Radiation + Anti-tumor drugs + Treg cells	NCT04678401	PHASE1
Leukemia	Treg cells	NCT01660607	PHASE1/2
Colorectal Cancer	Adaptive autologous cell immunotherapy	NCT00986518	PHASE1/2
Myeloid Leukemia in Remission	Recombinant WT1 Antigen-Specific Cancer Immunotherapeutic	NCT01513109	PHASE1/2
Myelofibrosis	CK0804	NCT05423691	PHASE1

DLI, donor lymphocyte infusion; UCB, Umbilical Cord Blood.

### 5.3 Precisely modulating the treatment of COVID-19 by targeting FOXP3

Despite the attainment of herd immunity, COVID-19 continues to pose a significant threat to human health owing to its escalating transmissibility and potential consequences on mortality rates. In the ongoing battle against COVID-19, it has been established that the treatment strategy primarily involves two components: early antiviral defense to facilitate virus clearance and late inhibition of inflammatory organ damage (**Figure 5**). Recent advancements in science have led to the development of small molecule antiviral drugs such as nematete/velitonavir and synacht/velitonavir, which have demonstrated promising results in clearing viruses (215, 216). However, as these drugs do not have a direct virucidal effect, their efficacy in severe patients remains suboptimal (217). Moreover, certain studies suggest that COVID-19-induced Tregs can exhibit characteristics akin to tumor-associated Tregs, leading to excessive suppression of the host’s antiviral response (218).Therefore, early modulation of Tregs quantity and function may enhance the therapeutic potential of these agents. Moreover, prioritizing late suppression of inflammatory organ damage is crucial in combating COVID-19. In a mouse model of experimental acute lung injury, increased accumulation of Tregs in BALF mediates alleviation of lung injury by inducing neutrophil apoptosis, macrophage endocytosis, and reducing fibrocyte recruitment (219). Additionally, Tregs can repair alveolar epithelial cells damaged by COVID-19 infection by producing AREG while maintaining blood oxygen saturation levels for ARDS patients during later stages of illness besides inhibiting systemic organs’ inflammatory damage and reducing autoimmune disease incidence (91). Moreover, Tregs can also attenuate signal transduction in the CXCL12/CXCR4 axis to mitigate pulmonary fibrosis levels following injury (220). Given that hypoxia can induce the activation of hypoxia-inducible factor-1α (HIF-1α) within the pulmonary microenvironment, resulting in subsequent proteasome-mediated degradation of FOXP3 and thereby impeding Tregs differentiation, oxygen inhalation emerges as a pivotal therapeutic intervention for severe COVID-19 (221). Apart from the low dose IL-2 treatment mentioned above, epigenetic strategies based on FOXP3+Tregs should not be overlooked as they can expand the number of Tregs while stabilizing their normal suppressor function. The current clinical utilization of dexamethasone, tocilizumab, chloroquine, and azithromycin can restore immune system homeostasis by upregulating FOXP3 expression in Tregs (16–18). Among them, tocilizumab is an anti-IL-6R monoclonal antibody (mAb), which has demonstrated clinical efficacy as an anti-COVID-19 drug by targeting the epigenetic inheritance of FOXP3+Tregs (222). Tocilizumab exerts its effects by inhibiting IL-6-induced Notch4 induction, thereby relieving Notch4-mediated inhibition on FOXP3 and AREG expression (222). This promotes alveolar epithelial cell repair, suppresses fibrosis progression, and mitigates the detrimental inflammatory cytokine storm triggered by the novel coronavirus. However, the amplification of Tregs alone did not yield the anticipated efficacy, possibly due to the SARS-CoV-2’s ability to impede Tregs recruitment in the respiratory tract during circulation (223). Interestingly, researchers have discovered a notable gender disparity in SARS-CoV-2 infection rates, with women exhibiting a lower susceptibility compared to men. This phenomenon may potentially be attributed to the presence of the *Foxp3* gene encoded on the X chromosome, whereby females possessing two X chromosomes demonstrate elevated levels of Tregs despite one being inactive (224). Paradoxically, autoimmune diseases exhibit a higher incidence in women rather than a lower one. Therefore, based on these aforementioned findings, further comprehensive investigations into Tregs are urgently warranted for a deeper understanding of their role in COVID-19 pathogenesis.

## 6 Conclusion

Tregs are a potent cell type capable of inducing self-tolerance, suppressing excessive immune system activation, maintaining pathogen defense, and performing various roles in different physiological settings. Extensive research has demonstrated that these functions are closely associated with the normal expression of FOXP3. In the presence of FOXP3, Tregs can secrete inhibitory cytokines such as IL-10 and IL-35 or inhibit the function of CD4+effector T cells through competitive consumption of IL-2. Moreover, granzyme and perforin-mediated cytotoxic mechanisms along with adenosine production induced by extracellular enzymes play a pivotal role in regulating immune homeostasis mediated by Tregs. Unlike the mechanisms above, Th-Tregs exhibit distinct differentiation patterns to precisely modulate immune function *in vivo*. Epigenetic regulation of Tregs, particularly involving *FOXP3* methylation, histone modifications, and post-translational modifications, plays a crucial role in maintaining lineage consistency among Tregs while regulating their function and enhancing clinical efficacy. Increasing evidence suggests that apart from their “classical” functions, FOXP3+Tregs also participate in processes such as stem cell maintenance, metabolic regulation, tissue repair and regeneration. Identifying these molecular mediators and pathways will significantly impact future advancements in regenerative medicine. Current preclinical and clinical studies are actively investigating therapeutic strategies targeting FOXP3+Tregs, specifically UP-Treg and DOWN-Treg, for the treatment of autoimmune diseases, tumors, infectious diseases (including COVID-19), improvement of islet β cell function in diabetes patients, and promotion of tissue repair. Notably, low-dose IL-2 has emerged as a clinically successful strategy for UP-Treg treatment. In the recent COVID-19 epidemic, scientists have demonstrated certain therapeutic effects using early DOWN-Treg and late UP-Treg strategies. Additionally, AREG produced by FOXP3+Tregs can facilitate the repair of alveolar epithelial cells damaged by COVID-19 infection while maintaining blood oxygen saturation levels in ARDS patients during later stages of the disease. However, due to the complex mechanism of Tregs immune regulation, limited precision of most epigenetic modulators, and incomplete understanding of Treg’s role in immune regulation, achieving ideal therapeutic effects based on Tregs epigenetics remains challenging. Therefore, precise and stable regulation of FOXP3+Tregs still poses an urgent challenge for future research. In this context, integration of emerging technologies such as externally controllable switches (e.g., light-activated Treg-scavenger antibodies) or CRISPR-dCAS9 with epigenetic manipulation tools holds promise for precise and sustained regulation of FOXP3+Tregs.

## Author contributions

YY: Writing – original draft. YR: Writing – review & editing. CL: Writing – review & editing. PL: Writing – review & editing. GZ: Writing – review & editing.

## Glossary

**Table:**

AD	Autoimmune Diseases
α-KG	α-ketoglutarate
AKT-mTOR	Protein Kinase B-mammalian Target of Rapamycin
AMPK	AMP-activated Protein Kinase
ARDS	Acute Respiratory Distress Syndrome
AREG	Amphiregulin
ATII	Type II Alveolar Epithelium Cell
ATRA	all-trans retinoic acid
BATF	Basic Leucine Zipper ATF-like Transcription Factor
BM-Tregs	Bone Marrow Tregs
CBP	CREB-binding Protein
CDK2	Cyclin-Dependent Kinase 2
CNS	Conserved Non-coding Sequence
CoREST	RE1-Silencing Transcription Factor Corepressor
COX-2	Cyclooxygenase-2
CREB/ATF	cyclic AMP response element binding protein/activating transcription factor
CRISPR/dCas9	Clustered Regularly Interspaced Short Palindromic Repeats Associated Death Cas9
CTLA4	Cytotoxic T-Lymphocyte Antigen 4
DNMTs	DNA Methyltransferases
DPPA3	Developmental Pluripotency-Associated 3
EGFR	Epidermal Growth Factor Receptor
FAO	Fatty Acid Oxidation
FOXP3	Forkhead Box P3
GLUT4	Glucose Transporter 4 Expression
HATs	Histone Acetyltransferases
HDACs	Histone Deacetylases
HSCs	Hematopoietic Stem Cells
HSP70	Heat Shock Protein 70
IBD	Inflammatory Bowel Disease
IL-2Rα	Interleukin-2 Receptor Alpha
IPEX syndrome	Immune Dysregulation, Polyendocrinopathy, Enteropathy, X-linked Syndrome
NIR	Near-infrared
NLK	Nemo-Like Kinase
NTF3	Neurotropin-3
nTreg	natural Treg
oxi-mCs	Oxidized-methylcytosines
OXPHOS	Oxidative Phosphorylation
PP1	Protein Phosphatase 1
PP6	Protein Phosphatase 6
PRMTs	Protein arginine methyltransferases
RA	Rheumatoid arthritis
RNF31	Ring Finger Protein 31
SEs	Super Enhancers
SIRT1	Sirtuin 1
SLE	Systemic Lupus Erythematosus
STAT	Signal Transducer and Activator of Transcription
STUB1	Stip1 Homology and U-Box Containing Protein 1
S1P	Sphingosine-1-phosphate
TALEs	Transcription Activator-like Proteins
Tconv cells	T conventional cells
TCR	T-Cell Receptor
TCR-CDK	T Cell Receptor-Cyclin-Dependent Kinase
TDG	Thymine DNA Glycosylase
TET	Ten-eleven Translocation
Th-Treg	Th-like Treg
TIP60	Tat-interactive Protein 60
TI-Tregs	Tumor Infiltrating Regulatory T cells
TME	Tumor Microenvironment
TRAF6	TNF Receptor-Associated Factor 6
Tregs	Regulatory T cells
TSDRs	Treg cell-Specific Demethylation Region
UHRF1	Ubiquitin-like with PHD and RING Finger Domains 1
USP	Ubiquitin-Specific Protease
ZF	Zinc-Finger
5mC	5-methylcytosine
5fmC	5-formylmethylcytosine.
